# Treatment of critical bleeding events in patients with immune thrombocytopenia: a protocol for a systematic review and meta-analysis

**DOI:** 10.1186/s13643-023-02436-6

**Published:** 2024-01-06

**Authors:** Emily Sirotich, Hasmik Nazaryan, Saifur Rahman Chowdhury, Gordon Guyatt, Arnav Agarwal, Russell Leong, Aaron Wen, Emily Xu, Bonnie Liu, Sushmitha Pallapothu, Preksha Rathod, Henry Y. Kwon, Jared Dookie, Amirmohammad Shafiee, Jay Charness, Jennifer DiRaimo, Dale Paynter, Barbara Pruitt, Gail Strachan, Rachel Couban, Zhikang Ye, Donald M. Arnold

**Affiliations:** 1 Michael G. DeGroote Centre for Transfusion Research, McMaster University, Hamilton, ON Canada; 2 Department of Health Research Methods, Evidence, and Impact, McMaster University, Hamilton, ON Canada; 3 Department of Medicine, McMaster University, Hamilton, ON Canada; 4 Faculty of Health Sciences, McMaster University, Hamilton, ON Canada; 5 Department of Surgery, Henry Ford Health Systems, Detroit, MI USA; 6 Platelet Disorder Support Association (PDSA), Cleveland, OH USA; 7 Faculty of Medicine, University of Ottawa, Ottawa, Canada

**Keywords:** Immune thrombocytopenia, Thrombocytopenia, Emergency management, Critical bleeding

## Abstract

**Background:**

Critical bleeding events in adults and children with ITP are medical emergencies; however, evidence-based treatment protocols are lacking. Due to the severe thrombocytopenia, (typically platelet count less than 20 × 10^9^/L), a critical bleed portends a high risk of death or disability. We plan to perform a systematic review and meta-analysis of treatments for critical bleeding in patients with ITP that will inform evidence-based recommendations.

**Methods:**

Literature searches will be conducted in four electronic databases: Ovid MEDLINE, Embase, Cochrane Central Register of Controlled Trials (CENTRAL), and PubMed. Eligible studies will be randomized controlled trials or observational studies that enrolled patients with ITP describing one or more interventions for the management of critical bleeding. Title and abstract screening, full-text screening, data extraction, and risk of bias evaluation will be conducted independently and in duplicate using Covidence and Excel. Outcomes will be pooled for meta-analysis where appropriate or summarized descriptively. Grading of Recommendations Assessment, Development, and Evaluation (GRADE) methodology will be used to evaluate the certainty of the evidence. Primary outcomes of interest will include frequency of critical bleeds, mortality and bleeding-related mortality, bleeding resolution, platelet count, and disability.

**Discussion:**

Evidence-based treatments for critical bleeding in patients with ITP are needed to improve patient outcomes and standardize care in the emergency setting.

**Systematic review registration:**

CRD42020161206.

**Supplementary Information:**

The online version contains supplementary material available at 10.1186/s13643-023-02436-6.

## Introduction

### Rationale

Immune thrombocytopenia (ITP) is an autoimmune bleeding disorder characterized by platelet counts below 100 × 10^9^/L in the absence of other causes [1, 2]. The disease affects both children and adults and has a female predominance [2, 3]. Although the majority of bleeding complications related to ITP are minor with no lasting effects, major bleeding episodes, especially in patients with severe thrombocytopenia (platelet counts below 20 × 10^9^/L) can lead to significant morbidity and mortality [4]. Pooled estimates of the 5-year risk of death from bleeding in ITP are as high as 47.8% for patients > 60 years [5]. For children, the risk of severe bleeding is approximately 3% [6, 7]. Intracranial hemorrhage (ICH) is the most severe type of bleeding event in patients with ITP. The incidence of ICH is 1.1% ± 0.1% for adults and 0.7% ± 0.1% for children [8]. The case fatality rate of ICH in children is approximately 25% [8].

Critical bleeds are defined as (i) a bleed in a critical anatomical site including intracranial, intraspinal, intraocular, retroperitoneal, pericardial, or intramuscular with compartment syndrome; or (ii) an ongoing bleed that results in hemodynamic instability or respiratory compromise [9]. Critical bleeds require urgent multimodal treatment in the emergency department or the in-patient setting with the goal of rapidly raising platelet counts and achieving hemostasis [10]. Acute management of a critical bleed might include typically ITP treatments such as intravenous immune globulin (IVIG) and corticosteroids, plus additional treatments including platelet transfusions [10, 11], antifibrinolytic medications [12], recombinant factor VIIa [13], urgent splenectomy, and thrombopoietin (TPO) receptor agonists, alone or in combination [1, 14–18].

### Objectives

Evidence-based guidelines for the management of a critical bleed in patients with ITP are lacking. Broad ITP treatment guidelines from the American Society of Hematology (ASH), published in 2019, did not address the management of critical bleeding [19]. We will conduct a systematic review and meta-analysis of treatments for adults and children, with the goal of informing the development of evidence-based guidelines.

## Methods

The protocol for this systematic review was developed based on the Preferred Reporting Items for Systematic Reviews and Meta-Analyses Protocols (PRISMA-P) (see Additional files for checklist) [18], and registered in PROSPERO (CRD42020161206). The systematic review will be reported in accordance with PRISMA guidelines.

### Eligibility criteria

We will include clinical trials and observational studies that enrolled patients who have suspected or confirmed ITP and critical bleeding. Studies must include a description or evaluation of one or more interventions, alone or in combination, with or without a comparator. We will use the ISTH definition of critical bleeding: (i) a bleed in a critical anatomical site including intracranial, intraspinal, intraocular, retroperitoneal, pericardial, or intramuscular with compartment syndrome; or (ii) an ongoing bleed that results in hemodynamic instability or respiratory compromise [9]. Given the recognized difficulties in establishing the diagnosis of ITP among patients with thrombocytopenia, we anticipate a high case mix in primary ITP studies. For this systematic review, we will include studies that enrolled at least 80% of ITP patients, or studies that reported data separately for patients with ITP.

### Information sources

With the aid of a medical librarian, searches will be conducted in four electronic databases from inception to 2023: Ovid MEDLINE, Embase, PubMed, and the Cochrane Central Register of Controlled Trials (CENTRAL).

### Search strategy

A combination of keywords and medical subject heading (MeSH) terms will be used: “Purpura, Thrombocytopenic, Idiopathic; Epidemiologic Studies, and Randomized Controlled Trial” (see Additional files for search strategies). No restrictions will be applied to language and publication status. Citation lists of identified reviews and primary publications will be screened for additional studies.

### Selection process

Reviewers will work independently and in pairs to conduct title and abstract screening and full-text reviews using pre-defined eligibility criteria and standardized forms. Disagreements will be resolved by a third reviewer.

### Data collection process

Reviewers will conduct abstractions independently and in duplicate using standardized forms. Discrepancies will be resolved by consensus, with input from a third reviewer if needed. For missing data, reviewers will attempt to contact study authors when possible.

### Data items

Any study data relevant to the research questions outlined above will be collected. The following study data will be abstracted from each study:

- Study citation and author contact details,
- Study design, duration, and setting,
- Country,
- Number of participants,
- Demographics (age, sex, comorbidities),
- ITP diagnoses of participants (primary, secondary, chronic, etc.),
- Intervention details (name, dose, frequency, etc.),
- Reported outcomes according to the outcomes of interest (outcome definitions, time-points collected, unit of measurement, sample size, statistical significance testing); and
- Funding sources.

### Outcomes and prioritization

Outcome assessments will be restricted to 7 days or the duration of hospitalization since the objective is to describe the acute management of critical bleeds. Along those lines, we focused this review on interventions that typically have a rapid or immediate effect on increasing platelet counts or restoring hemostasis, including corticosteroids, intravenous immunoglobulin, anti-D immunoglobulin, platelet transfusion, tranexamic acid, TPO receptor agonists (romiplostim, eltrombopag, avatrombopag), recombinant factor VIIa, and urgent splenectomy. Outcomes of interest are mortality (all-cause and bleeding-related), resolution of bleeding, disability, platelet counts, platelet count responses (minimal, > 30 × 10^9^/L; overall, > 50 × 10^9^/L) [20], new onset of bleeding, duration of hospital stay, need for and duration of intensive care unit admission, and treatment-related adverse events.

### Risk of bias in individual studies

Risk of bias will be assessed using a revision of the Cochrane Risk of Bias version 2.0 assessment tool for randomized trials [21], and the ROBINS-I for Risk of Bias Assessment for observational studies [22]. Risk of bias will be classified as ‘‘low’’, ‘‘some concerns-probably low’’, ‘some concerns-probably high’’, or ‘‘high’’ for the following domains: bias due to randomization, bias due to deviations from the intended intervention, bias due to missing outcome data, bias in the measurement of the outcome, bias in the selection of the reported result, and other biases [23]. We will rate the overall risk of bias as the highest risk attributed to any criterion.

Reviewers will conduct abstractions and risk of bias assessments independently and in duplicate using standardized forms. Discrepancies will be resolved by consensus, with input from a third reviewer if needed.

### Data synthesis and summary measures

We anticipate that there will be limited direct evidence available from published reports and significant heterogeneity between study types, patients, interventions evaluated, and outcomes which may preclude statistical meta-analysis. In that case, we will summarize study findings descriptively and provide aggregate results per intervention and outcome where appropriate. Specifically, we will describe the anatomical sites of bleeding, the interventions, and the outcomes for the participants. If meta-analysis is feasible, we will present continuous outcomes as mean differences, dichotomous outcomes as risk ratios, and time-to-event data (as reported by the authors) as hazard ratios, all with 95% CIs. We will assume a normal distribution for continuous outcomes and will convert the median to mean and interquartile ranges to standard deviations (SD) as per guidance from the Cochrane Collaboration [24]. When feasible, we will combine RCTs and observational studies to perform quantitative analyses and present results in single pooled estimates. We will also present results from RCTs and observational studies separately in subsequent subgroup analysis.

We will group the interventions according to the following criteria to explore which factors might affect the effectiveness of critical bleeding interventions:

- Interventions: corticosteroids, intravenous immunoglobulin, anti-D immunoglobulin, platelet transfusion, tranexamic acid, TPO receptor agonists (romiplostim, eltrombopag, avatrombopag), recombinant factor VIIa, and urgent splenectomy;
- Dosage and number of treatments;
- Duration of ITP diagnosis; and
- Age of patient population.

### Statistical analysis and meta-biases

Effect measures will be reported according to the outcome per study. Due to the predicted limited number of eligible studies, descriptive statistics will be used to summarize treatment effects in each study by appropriate subpopulation. Where a meta-analysis is possible, we will pool the results using DerSimonian–Laird random-effects models to account for variation in effect size amongst studies. We will report pooled estimates with 95% CIs, with two-sided *p*-values for each meta-analyzed outcome. If a meta-analysis is performed, heterogeneity among included studies will be quantified using inconsistency index (*I*^2^) and *p* values from the chi-square test for homogeneity. The threshold for *I*^2^ value will be interpreted as follows: 0–40% represents minimal heterogeneity, 30–60% represents moderate heterogeneity, 50–90% represents substantial heterogeneity, and 75–100% represents considerable heterogeneity [25]. For homogeneous studies, we will present forest plots with or without pooled estimates. In the case of ≥ 10 studies included in the meta-analysis, we will assess publication bias using funnel plots [26]. Most of the ITP treatment trials are small-scale trials. When studies report missing data (loss to follow-up), we will conduct a complete case analysis as our primary analysis.

In addition to the meta-analyses, we will present a structured narrative synthesis, and results will be presented in structured tables and figures organized by treatment type. All analyses will be performed using R (version 4.2.3). We will use the MAGIC Authors Publishing Platform (https://app.magicapp.org) to generate the GRADE summary of the findings table.

### Subgroup analysis and sensitivity analysis

We will perform subgroup analysis for risk of bias assessment. Subgroup analyses will be performed when two or more studies are in a given subgroup. Since we anticipate that many studies will be at high risk of bias, we will conduct subgroup analyses based on the risk of bias judgements (high risk of bias versus low risk of bias) and consider that high risk of bias studies may exaggerate treatment effects. A subgroup analysis with RCTs and observational studies will also be completed. Regardless of the observed statistical heterogeneity, we will also conduct the following prespecified subgroup analyses when each subgroup was represented by at least two studies: age (children vs. adults), and type of ITP (newly diagnosed, persistent, and chronic). If we have the required number of studies to perform subgroup analysis, we will conduct tests of interaction to establish whether the subgroups differed significantly from each other [27]. We will perform univariate meta-regressions to assess the effects of the participant’s age and type of ITP (newly diagnosed, persistent, and chronic) on the intervention effects. Additionally, we will perform sensitivity analysis using fixed-effects models to assess the differences in alternative methods.

### Confidence in cumulative evidence

We will consider the overall certainty in evidence for each outcome using the GRADE framework, based on the following domains: risk of bias, imprecision, inconsistency, indirectness, and publication bias [23]. Overall certainty of evidence will be rated as very low, low, moderate, or high. Summary of findings tables will be used to outline the certainty of the body of evidence for each outcome. All decisions to downgrade the certainty of evidence will be included in the footnotes of the summary of findings tables. A summary of the evidence will also be presented. We will follow the GRADE framework to integrate randomized and non-randomized studies using ROBINS-I into the evidence tables [24]. We will consider rating down the certainty of evidence for risk of bias based on lack of blinding for subjective outcomes only.

We will make judgments of imprecision using a minimally contextualized approach. We will consider any CI encompassing the null effect to be imprecise, with consideration of important and trivial effect. Due to the rarity of the disease and the small population of interest, there may be heterogeneity across the studies in terms of populations and/or treatments. There may be true differences in the underlying treatment effect due to this limited sample size and heterogeneity of the treatments. We will explore explanations for heterogeneity and downgrade the certainty of evidence accordingly. If classifying patients into easily identifiable subpopulations is feasible, effect measures will be reported across subpopulations rather than downgrading the certainty of evidence for inconsistency in effect size. Two people with experience in using GRADE will rate the certainty of the evidence for each comparison separately and resolve discrepancies by consensus.

## Discussion

This systematic review aims to identify effective treatments for patients with ITP and critical bleeding. The outcomes of interest reflect clinical practice and relevant clinical outcomes including platelet count levels, mortality, disability, and hospital length of stay. The results of this systematic review will be used to inform evidence-based guidelines for the management of ITP patients with critical bleeding.

This review has potential limitations. We anticipate that the primary studies included in this review will have heterogeneous designs, with critical bleed outcomes being reported mainly in case series or observational studies and having minimal reporting in randomized trials. Since the definition of critical bleeds in ITP patients was recently standardized, studies that report bleeding according to this definition will not be available. Therefore, comparing the various definitions of bleeding across studies may limit the interpretability of bleeding results. Lastly, the availability of randomized trials that evaluate interventions during a critical bleed will be limited because of the urgent nature of the event.

The identification of optimal treatment and management strategies for ITP bleeding emergencies will have immediate implications for patients and providers. This information will be used by hematologists and physicians in the emergency department when faced with this rare but life-threatening event.

### Protocol amendments

Any amendments to this protocol in the carrying out of this systematic review will be documented and reported in both the PROSPERO register and any subsequent publications.

### Dissemination plans

The findings of this systematic review will be disseminated through publication in peer-reviewed journals and via relevant conferences. In addition, the results will also be shared with potential stakeholders, including the Platelet Disorder Support Association and the Canadian Institutes for Health Research.

### Supplementary Information

**Additional file 1:** PRISMA-P Checklist (pdf). This checklist addresses the recommended items to report in a systematic review protocol and references their locations within the text.

**Additional file 2:** Search Strategy (pdf). The search strategy developed for MEDLINE (Ovid) will be modified for use in other databases.

## Data Availability

All data and materials were accessed through institutional library domains.

## Abbreviations

ITP: Immune thrombocytopenia
CENTRAL: Cochrane Central Register of Controlled Trials
CI: Confidence interval
GRADE: Grading of Recommendations Assessment, Development and Evaluation
PRISMA: Preferred Reporting Items for Systematic Review and Meta-Analysis
PRISMA-P: Preferred Reporting Items for Systematic Review and Meta-Analysis Protocols
RCT: Randomized controlled trial

## References

[1] Neunert C, Lim W, Crowther M, Cohen A, Solberg L, Crowther MA (2011). The American Society of Hematology 2011 evidence-based practice guideline for immune thrombocytopenia. Blood.

[2] Segal J, Powe N (2006). Prevalence of immune thrombocytopenia: Analyses of administrative data. J Thromb Haemost.

[3] Piel-Julian M-L, Mahévas M, Germain J, Languille L, Comont T, Lapeyre-Mestre M (2018). Risk factors for bleeding, including platelet count threshold, in newly diagnosed immune thrombocytopenia adults. J Thromb Haemost.

[4] Terrell DR, Beebe LA, Neas BR, Vesely SK, Segal JB, George JN (2012). Prevalence of primary immune thrombocytopenia in Oklahoma. Am J Hematol.

[5] Cohen YC, Djulbegovic B, Shamai-Lubovitz O, Mozes B (2000). The bleeding risk and natural history of idiopathic thrombocytopenic purpura in patients with persistent low platelet counts. Arch Intern Med.

[6] Kühne T, Buchanan GR, Zimmerman S, Michaels LA, Kohan R, Berchtold W, Imbach P (2003). A prospective comparative study of 2540 infants and children with newly diagnosed idiopathic thrombocytopenic purpura (ITP) from the Intercontinental Childhood ITP Study Group. J Pediatr.

[7] Mithoowani S, Cervi A, Shah N, Ejaz R, Sirotich E, Barty R (2020). Management of major bleeds in patients with immune thrombocytopenia. J Thromb Haemost.

[8] Bhatt P, Yagnik PJ, Ayensu M, Khan AW, Adjei A, Parmar N (2020). Temporal Trends of Intracranial Hemorrhage Among Immune Thrombocytopenia Hospitalizations in the United States. Cureus.

[9] Sirotich E, Guyatt G, Gabe C, Ye Z, Beck CE, Breakey V (2021). Definition of a critical bleed in patients with immune thrombocytopenia: Communication from the ISTH SSC Subcommittee on Platelet Immunology. J Thromb Haemost.

[10] Salama A, Kiesewetter H, Kalus U, Movassaghi K, Meyer O (2008). Massive platelet transfusion is a rapidly effective emergency treatment in patients with refractory autoimmune thrombocytopenia. Thromb Haemost.

[11] Bartholomew JR, Salgia R, Bell WR (1989). Control of bleeding in patients with immune and nonimmune thrombocytopenia with aminocaproic acid. Arch Intern Med.

[12] Salama A, Rieke M, Kiesewetter H, von Depka M (2009). Experiences with recombinant FVIIa in the emergency treatment of patients with autoimmune thrombocytopenia: a review of the literature. Ann Hematol.

[13] Provan D, Stasi R, Newland AC, Blanchette VS, Bolton-Maggs P, Bussel JB (2010). International consensus report on the investigation and management of primary immune thrombocytopenia. Blood.

[14] Arnold DM (2015). Bleeding complications in immune thrombocytopenia. Hematology Am Soc Hematol Educ Program.

[15] Mayer B, Salama A (2017). Successful treatment of bleeding with tranexamic acid in a series of 12 patients with immune thrombocytopenia. Vox Sang.

[16] Bavunoğlu I, Eşkazan AE, Ar MC, Cengiz M, Yavuzer S, Salihoğlu A (2016). Treatment of patients with immune thrombocytopenia admitted to the emergency room. Int J Hematol.

[17] Parodi E, Giordano P, Rivetti E, Giraudo MT, Ansaldi G, Davitto M (2014). Efficacy of combined intravenous immunoglobulins and steroids in children with primary immune thrombocytopenia and persistent bleeding symptoms. Blood Transfus.

[18] Neunert C, Terrell DR, Arnold DM, Buchanan G, Cines DB, Cooper N (2019). American Society of Hematology 2019 guidelines for immune thrombocytopenia. Blood Adv.

[19] Shamseer L, Moher D, Clarke M, Ghersi D, Liberati A, Petticrew M (2015). Preferred reporting items for systematic review and meta-analysis protocols (PRISMA-P) 2015: elaboration and explanation. BMJ.

[20] Rodeghiero F, Michel M, Gernsheimer T, Ruggeri M, Blanchette V, Bussel JB (2013). Standardization of bleeding assessment in immune thrombocytopenia: Report from the International Working Group. Blood.

[21] Sterne JAC, Savović J, Page MJ, Elbers RG, Blencowe NS, Boutron I (2019). RoB 2: A revised tool for assessing risk of bias in randomized trials. BMJ.

[22] Sterne JAC, Hernán MA, Reeves BC, Savović J, Berkman ND, Viswanathan M (2016). ROBINS-I: A tool for assessing risk of bias in non-randomized studies of interventions. BMJ.

[23] Schünemann H, Brożek J, Guyatt G, Oxman A. (Eds.). GRADE handbook for grading quality of evidence and strength of recommendations (Updated October 2013). The GRADE Working Group. 2013.

[24] Higgins JPT, Thomas J, Chandler J, Cumpston M, Li T, Page MJ, Welch V A. (Eds.). Cochrane Handbook for Systematic Reviews of Interventions version 6.4 (Updated August 2023). Cochrane. 2023.

[25] Deeks JJ, Higgins JPT, Altman DG. (Eds.). Chapter 10: Analysing data and undertaking meta-analyses. Cochrane Handbook for Systematic Reviews of Interventions version 6.4 (Updated August 2023). Cochrane. 2023.

[26] Sterne JAC, Sutton AJ, Ioannidis JP (2011). Recommendations for examining and interpreting funnel plot asymmetry in meta-analyses of randomized controlled trials. BMJ.

[27] Altman DG, Bland JM (2003). Interaction revisited: The difference between two estimates. BMJ.

