# Supplementary material for: Treatment of critical bleeding events in patients with immune thrombocytopenia: a protocol for a systematic review and meta-analysis
Source: Syst Rev. 2024 Jan 6;13:21. doi: 10.1186/s13643-023-02436-6 (PMC10770981; doi:10.1186/s13643-023-02436-6)
Supplement: Supplementary file 2 — Additional file 2: Search Strategy (pdf). The search strategy developed for MEDLINE (Ovid) will be modified for use in other databases. [file 13643_2023_2436_MOESM2_ESM.doc]

Summary of search and strategies – ITP March 11, 2020

| MEDLINE | 5984 |
| --- | --- |
| EMBASE | 7146 |
| Central | 995 |
| PubMed | 1916 |
| Subtotal | 16041 |
| -dupes | -3405 |
| Total | 12636 |

**Database:** OVID Medline Epub Ahead of Print, In-Process & Other Non-Indexed Citations (Ovid MEDLINE(R) Daily and Ovid MEDLINE(R) 1946 to Present)

**Search Strategy:**

1 Purpura, Thrombocytopenic, Idiopathic/ (6219)

2 ITP.mp. (6994)

3 ((idiopath* or immun* or autoimmun*) adj3 (thrombocytopen* or thrombo-

cytopen*)).mp. (13105)

4 werlhof*.mp. [mp=title, abstract, original title, name of substance word,

subject heading word, floating sub-heading word, keyword heading word, organism

supplementary concept word, protocol supplementary concept word, rare disease

supplementary concept word, unique identifier, synonyms] (301)

5 or/1-4 (15181)

Annotation: ITP block

6 randomized controlled trial.pt. (501343)

7 controlled clinical trial.pt. (93566)

8 randomized.ab. (472423)

9 placebo.ab. (205820)

10 drug therapy.fs. (2185180)

11 randomly.ab. (328804)

12 trial.ab. (497519)

13 groups.ab. (2020445)

14 or/6-13 (4653137)

Annotation: Cochrane HSSS RCT filter

15 (Randomized Controlled Trial or Controlled Clinical Trial or Pragmatic

Clinical Trial or Equivalence Trial or Clinical Trial, Phase III).pt. (593236)

16 Randomized Controlled Trial/ (501343)

17 exp Randomized Controlled Trials as Topic/ (134022)

18 "Randomized Controlled Trial (topic)"/ (0)

19 Controlled Clinical Trial/ (93566)

20 exp Controlled Clinical Trials as Topic/ (139239)

21 "Controlled Clinical Trial (topic)"/ (0)

22 Randomization/ (102249)

23 Random Allocation/ (102249)

24 Double-Blind Method/ (156428)

25 Double Blind Procedure/ (0)

26 Double-Blind Studies/ (156428)

27 Single-Blind Method/ (28191)

28 Single Blind Procedure/ (0)

29 Single-Blind Studies/ (28191)

30 Placebos/ (34751)

31 Placebo/ (0)

32 Control Groups/ (1658)

33 Control Group/ (1658)

34 (random* or sham or placebo*).ti,ab,hw,kf,kw. (1469526)

35 ((singl* or doubl*) adj (blind* or dumm* or mask*)).ti,ab,hw,kf,kw. (233336)

36 ((tripl* or trebl*) adj (blind* or dumm* or mask*)).ti,ab,hw,kf,kw. (978)

37 (control* adj3 (study or studies or trial* or group*)).ti,ab,kf,kw. (961925)

38 (Nonrandom* or non random* or non-random* or quasi-random* or

quasirandom*).ti,ab,hw,kf,kw. (43025)

39 allocated.ti,ab,hw. (64040)

40 ((open label or open-label) adj5 (study or studies or trial*)).ti,ab,hw,kf,kw.

(33713)

41 ((equivalence or superiority or non-inferiority or noninferiority) adj3 (study

or studies or trial*)).ti,ab,hw,kf,kw. (7917)

42 (pragmatic study or pragmatic studies).ti,ab,hw,kf,kw. (389)

43 ((pragmatic or practical) adj3 trial*).ti,ab,hw,kf,kw. (4834)

44 ((quasiexperimental or quasi-experimental) adj3 (study or studies or

trial*)).ti,ab,hw,kf,kw. (7507)

45 (phase adj3 (III or "3") adj3 (study or studies or trial*)).ti,hw,kf,kw.

(27481)

46 or/15-45 (2114506)

Annotation: CADTH CCT filter

47 Epidemiologic Studies/ (8235)

48 exp Case-Control Studies/ (1061386)

49 exp Cohort Studies/ (1964837)

50 Case control.tw. (122549)

51 (cohort adj (study or studies)).tw. (197278)

52 Cohort analy$.tw. (7749)

53 (Follow up adj (study or studies)).tw. (48569)

54 (observational adj (study or studies)).tw. (102444)

55 Longitudinal.tw. (238438)

56 Retrospective.tw. (512294)

57 Cross sectional.tw. (339614)

58 Cross-sectional studies/ (320256)

59 or/47-58 (2938516)

Annotation: SIGN Observational studies filter

60 14 or 46 or 59 (7117946)

61 animals/ not humans/ (4642996)

62 60 not 61 (6347796)

63 5 and 62 (5984)

**Database:** EMBASE <1974 to 2020 March 10>

**Search Strategy:**

1 idiopathic thrombocytopenic purpura/ (14293)

2 ITP.mp. (11010)

3 ((idiopath* or immun* or autoimmun*) adj3 (thrombocytopen* or thrombo-

cytopen*)).mp. (21810)

4 werlhof*.mp. (113)

5 or/1-4 (24094)

6 exp animals/ or exp invertebrate/ or animal experiment/ or animal model/ or

animal tissue/ or animal cell/ or nonhuman/ (27059885)

7 human/ or normal human/ or human cell/ (20702328)

8 6 and 7 (20639718)

9 6 not 8 (6420167)

10 5 not 9 (22697)

11 random:.tw. or placebo:.mp. or double-blind:.tw. (1762920)

12 ((treatment or control) adj3 group*).ab. (852999)

13 (allocat* adj5 group*).ab. (32306)

14 ((clinical or control*) adj3 trial).ti,ab,kw. (404761)

15 or/11-14 (2466991)

Annotation: modified HIRU RCT filter

16 10 and 15 (2176)

17 (Randomized Controlled Trial or Controlled Clinical Trial or Pragmatic

Clinical Trial or Equivalence Trial or Clinical Trial, Phase III).pt. (0)

18 Randomized Controlled Trial/ (593554)

19 exp Randomized Controlled Trials as Topic/ (175299)

20 "Randomized Controlled Trial (topic)"/ (175299)

21 Controlled Clinical Trial/ (463562)

22 exp Controlled Clinical Trials as Topic/ (182424)

23 "Controlled Clinical Trial (topic)"/ (10604)

24 Randomization/ (86167)

25 Random Allocation/ (82388)

26 Double-Blind Method/ (145847)

27 Double Blind Procedure/ (170229)

28 Double-Blind Studies/ (127818)

29 Single-Blind Method/ (36195)

30 Single Blind Procedure/ (38189)

31 Single-Blind Studies/ (38189)

32 Placebos/ (290937)

33 Placebo/ (347180)

34 Control Groups/ (110448)

35 Control Group/ (110448)

36 (random* or sham or placebo*).mp. (2011398)

37 ((singl* or doubl*) adj (blind* or dumm* or mask*)).mp. (301261)

38 ((tripl* or trebl*) adj (blind* or dumm* or mask*)).mp. (1305)

39 (control* adj3 (study or studies or trial* or group*)).mp. (8016522)

40 (Nonrandom* or non random* or non-random* or quasi-random* or

quasirandom*).mp. [mp=title, abstract, heading word, drug trade name, original title,

device manufacturer, drug manufacturer, device trade name, keyword, floating

subheading word, candidate term word] (54137)

41 allocated.mp. (82712)

42 ((open label or open-label) adj5 (study or studies or trial*)).mp. [mp=title,

abstract, heading word, drug trade name, original title, device manufacturer, drug

manufacturer, device trade name, keyword, floating subheading word, candidate term

word] (61524)

43 ((equivalence or superiority or non-inferiority or noninferiority) adj3 (study

or studies or trial*)).mp. (11832)

44 (pragmatic study or pragmatic studies).mp. [mp=title, abstract, heading word,

drug trade name, original title, device manufacturer, drug manufacturer, device trade

name, keyword, floating subheading word, candidate term word] (564)

45 ((pragmatic or practical) adj3 trial*).mp. [mp=title, abstract, heading word,

drug trade name, original title, device manufacturer, drug manufacturer, device trade

name, keyword, floating subheading word, candidate term word] (5188)

46 ((quasiexperimental or quasi-experimental) adj3 (study or studies or

trial*)).mp. [mp=title, abstract, heading word, drug trade name, original title,

device manufacturer, drug manufacturer, device trade name, keyword, floating

subheading word, candidate term word] (11837)

47 (phase adj3 (III or "3") adj3 (study or studies or trial*)).mp. [mp=title,

abstract, heading word, drug trade name, original title, device manufacturer, drug

manufacturer, device trade name, keyword, floating subheading word, candidate term

word] (121854)

48 or/17-47 (8800670)

Annotation: CADTH CCT filter

49 10 and 48 (5470)

50 clinical study/ (154759)

51 case control study/ (152869)

52 family study/ (25993)

53 longitudinal study/ (136670)

54 retrospective study/ (888509)

55 prospective study/ (585814)

56 cohort analysis/ (556646)

57 (Cohort adj (study or studies)).mp. (294384)

58 (Case control adj (study or studies)).tw. (130860)

59 (follow up adj (study or studies)).tw. (62364)

60 (observational adj (study or studies)).tw. (161004)

61 (epidemiologic$ adj (study or studies)).tw. (104626)

62 (cross sectional adj (study or studies)).tw. (209351)

63 or/50-62 (2626761)

Annotation: SIGN observational studies filter

64 10 and 63 (2868)

65 16 or 49 or 64 (7146)

66 10 and (15 or 48 or 63) (7146)

**Database:** Cochrane Library

**Search Strategy:**

Search Name: ITP

Date Run: 11/03/2020 21:11:27

ID Search Hits

#1 MeSH descriptor: [Purpura, Thrombocytopenic, Idiopathic] explode all trees 269

#2 (ITP):ti,ab,kw (Word variations have been searched) 654

#3 (idiopath* or immun* or autoimmun*) near/3 (thrombocytopen* or thrombo-cytopen*) 954

#4 (werlhof*):ti,ab,kw (Word variations have been searched) 0

#5 #1 or #2 or #3 or #4 in Trials 995

**Database:** PubMed

**Search Strategy:**

Search ((((publisher[sb] OR inprocess[sb] OR pubmednotmedline[sb] OR pubstatusaheadofprint)))) AND (((((((idiopath* or immune or autoimmune*) AND (thrombocytopen* or thrombo- cytopen*))))) OR ITP) OR werlhof*)

Sort by: PublicationDate 1916
